# From Chemistry to Behavior. Molecular Structure and Bioactivity of Repellents against *Ixodes ricinus* Ticks

**DOI:** 10.1371/journal.pone.0067832

**Published:** 2013-06-21

**Authors:** Simone Del Fabbro, Francesco Nazzi

**Affiliations:** Dipartimento di Scienze Agrarie e Ambientali, Università degli Studi di Udine, Udine, Italy; Washington State University, United States of America

## Abstract

Tick-borne zoonoses are considered as emerging diseases. Tick repellents represent an effective tool for reducing the risk of tick bite and pathogens transmission. Previous work demonstrated the repellent activity of the phenylpropanoid eugenol against *Ixodes ricinus*; here we investigate the relationship between molecular structure and repellency in a group of substances related to that compound. We report the biological activity of 18 compounds varying for the presence/number of several moieties, including hydroxyl and methoxy groups and carbon side-chain. Each compound was tested at different doses with a bioassay designed to measure repellency against individual tick nymphs. Both vapor pressure and chemical features of the tested compounds appeared to be related to repellency. In particular, the hydroxyl and methoxy groups as well as the side-chain on the benzene ring seem to play a role. These results are discussed in light of available data on chemical perception in ticks. In the course of the study new repellent compounds were identified; the biological activity of some of them (at least as effective as the “gold standard” repellent DEET) appears to be very promising from a practical point of view.

## Introduction

Ticks are obligate blood-feeding ectoparasites of great veterinary and public health importance. Ticks biting humans can cause severe allergic reactions, but the primary concern is their ability to transmit a number of disease-causing agents during the blood meals. For instance, in Europe, Lyme borreliosis and Tick-borne encephalitis, which are transmitted by the tick *Ixodes ricinus* (L.), are considered emerging diseases [1], [2].

Avoidance of tick-infested areas is the primary personal protection measure to prevent tick bites; however, in order to minimize the risk of tick contact, some simple preventive measures can be taken, such as a sensible behavior or appropriate clothing. Tick repellents and acaricides represent effective tools for reducing the risk of tick bite and pathogens transmission, before and after tick contact, respectively [3]. Tick repellent compounds in different commercial formulations are available and have been demonstrated to reduce the risk of bites when applied to clothing or bare skin (see for example [4]). Since its registration for commercial use, DEET (*N,N*-Diethyl-3-methyl-benzamide or *N,N*-Diethyl-*meta*-toluamide) has become the main active ingredient in most commercial insect- and tick-repellents used on human skin [4]. Although DEET and other common products based on synthetic pyrethroids are very effective and relatively safe repellents (for DEET safety see for example [5]–[7]), some concern exists about possible adverse effects on human health [8], [9] and people frequently perceive synthetic repellents as a potential source of toxicity [10]. Moreover, arthropods show differential responses to these products, indicating the possibility of adaptation and emerging resistance or insensitivity [11]–[14]. For these reasons the development of novel repellents could be of great value and so would be the discovery of new bioactive plant-derived compounds, which are generally more acceptable to people unwilling to use synthetic chemicals [4]. In this regard, a better knowledge about the molecular determinants of the biological activity of such substances may be very important from a practical point of view, in that it may allow a targeted development of new repellents or a faster screening of candidate natural compounds.

Preliminary experiments conducted in our laboratory suggested a biological activity of *Ocimum basilicum* L. (sweet basil) on *I. ricinus* [15], integrating previous data about the biological effects of this and related *Ocimum* species against other arthropods [16]–[20], including other tick species [21], [22]. The compound responsible for the repellency was later identified as eugenol using a bioassay assisted HPLC fractionation protocol followed by GC-MS identification [23]. Eugenol appeared to be as repellent as the reference substance DEET at the doses of 1000 and 100 µg; however, unlike eugenol, DEET was also active at the dose of 10 µg.

Several biological effects of eugenol on invertebrates have already been demonstrated; some examples are: pre- and postsynaptic effects on snail neurons [24]; insecticidal activity [25], [26]; acaricidal activity [27], [28]; repellent activity against insects [29]. Apart from our previous study, comments about a possible activity of eugenol on *I. ricinus* are also available [30], [31]. However, to our knowledge, no attempts have been made so far to correlate the chemical features of this substance to the repellency against any tick species.

Ecological interactions of ticks are largely mediated by chemical compounds that can affect several crucial stages of the life cycle [32], however the functional basis of their actions are not yet completely understood. In other arthropods a detailed knowledge of the functional basis of chemical mediated communication has been achieved thanks to the fruitful integration of several investigation techniques, including analytical chemistry, neurobiology, molecular biology, and most recent genomic approaches [33]. In the case of ticks, limited genomic data as well as reduced homology with other well studied arthropods has made the task particularly challenging. In fact, what is actually known about tick olfaction is confined to the important morphological and electrophysiological studies on the olfactory sensilla, that are concentrated in the Haller's organ, located on the tarsi of the first pair of legs [32], [34]–[38]. As regards further components of the olfactory system, neither olfactory receptors nor odorant-binding proteins (OBPs) have been described in ticks, while only 1 chemosensory protein (CSP) has been found, so far, in *Ixodes scapularis* Say [39].

For these reasons, the elucidation of the molecular basis of chemoreception in ticks needs to be pursued from a variety of indirect routes, including studies on the relationship between structure and activity of semiochemicals. Several studies on this relationship have been carried out on other arthropods using different approaches, either applying mathematical models to numerically express the chemical structure of the compounds or simply relating the presence of a given chemical feature to the repellency [40]–[43].

We investigated the molecular determinants of the repellent activity of eugenol and analyzed the factors accounting for such bioactivity. In view of this goal, several substances with similar structure were tested against *I. ricinus* nymphs and a graphical approach was used to investigate the relationship between structure and bioactivity. The study showed that certain combinations of chemical features are related to repellency; new effective repellent compounds were also identified. Some working hypotheses on the molecular basis of chemoreception in *I. ricinus* were drawn from the results.

## Materials and Methods

### Ethics statement

The bioassays were carried out using *I. ricinus* nymphs collected from May to November by dragging in mixed woodlands and ecotones in Friuli Venezia Giulia (north-eastern Italy). *I. ricinus* is not an endangered or protected species; therefore, according to local regulations, no specific permissions were required either for collecting the specimens employed in the experimental work or for the field sampling in private properties.

### Ticks used in this study

Nymphs were used for this study as this is the most important developmental stage from the epidemiological point of view, being both abundant in the environment and active in human biting and pathogen transmission.

Collected nymphs were kept inside sealed polypropylene tubes, in which a high relative humidity was provided by a damp strip of filter paper, and were maintained at room temperature in the dark until using in the assays. Most ticks were tested within 3 weeks after collection. In any case, only ticks that appeared to be active in the storing tubes were used for the bioassays. Each tick was used only once.

### Bioassay

**Figure 1:**
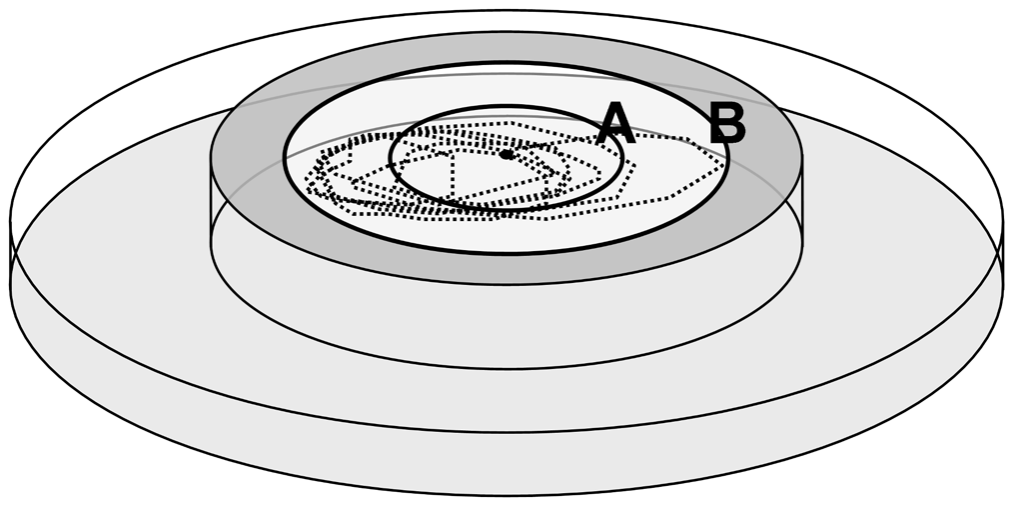
Glass arena used in the bioassays. A 6 cm diameter glass Petri dish was placed upside down in a larger Petri dish containing a wet piece of filter paper (light grey). Two concentric circles were drawn on the inner surface of the Petri dish, having 1 cm radius (start line or line A) and 2 cm radius (finish line or line B). The treatment was applied with a pipette outside the finish line (dark grey) on the outer surface of the Petri dish in 100 µl volume of solvent. After the complete evaporation of the solvent, a single nymph was placed with a fine paint brush in the center of the arena and the time spent to go from line A over line B was recorded. The dashed line represents the track captured from the video of a single tick moving on an arena treated with a repellent substance (in this case 100 µg of eugenol).

To test the effect of different stimuli on the behavior of ticks, a simple lab bioassay was used that was described previously [15], [23]. Briefly, a circular arena was obtained placing upside down a 6 cm diameter glass Petri dish. Two concentric circles were drawn on the inner surface of the Petri dish, having 1 cm radius (start line or line A) and 2 cm radius (finish line or line B). The treatment was applied with a pipette outside the finish line on the outer surface of the Petri dish in 100 µl volume of solvent (acetone). After the complete evaporation of the solvent, the arena was placed on a wet piece of filter paper inside a larger Petri dish (Figure 1); in this way a positive humidity gradient was created encouraging the centrifugal movement of the tick. A single nymph was placed with a fine paint brush in the center of the arena and carefully observed throughout the experiment; the time spent to go from line A over line B was recorded.

Under these conditions, in presence of non-repellent stimuli the nymph simply goes straight from the center of the arena to the edge of it, whereas if a repellent substance is used, the tick walks towards the finish line, turning before reaching the treated area.

If the start line was not crossed before 45 seconds, the nymph was discarded; if after 3.5 minutes the tick did not cross the finish line, 210 seconds was recorded as the finish time.

### Tested substances

Several compounds differing from eugenol for 1 or more molecular features were tested. In particular, since eugenol consists of a hydroxyl group (–OH), a methoxy group (–O–CH_3_) and an allyl side-chain (–CH_2_–CH = CH_2_) on a benzene ring, commercially available substances in which the hydroxyl and methoxy groups were maintained, eliminated or replaced with a methoxy or a hydroxyl group respectively were tested as well as compounds with an additional methoxy group. The allyl side-chain was maintained, eliminated or replaced with a methyl, ethyl or vinyl side-chain. Tested substances are reported in Table 1, along with eugenol. Pure compounds were purchased from Sigma, Aldrich, Sigma-Aldrich and SAFC.

**Table 1:** List of the substances considered in this study.

Abbr	CAS N°	Substance name (and synonyms)	Differences from eugenol
EUG	97-53-0	4-Allyl-2-methoxyphenol (4-Allylguaiacol; Eugenol)	-
SYR	91-10-1	2,6-Dimethoxyphenol (Syringol)	no side-chain + extra methoxy group
ADP	6627-88-9	4-Allyl-2,6-dimethoxyphenol (2,6-Dimethoxy-4-allylphenol)	extra methoxy group
CAT	120-80-9	1,2-Benzenediol (Catechol; 1,2-Dihydroxybenzene)	no side-chain + hydroxyl instead of methoxy group
MCA	452-86-8	4-Methyl-1,2-benzenediol (4-Methylcatechol)	methyl side-chain + hydroxyl instead of methoxy group
ECA	1124-39-6	4-Ethyl-1,2-benzenediol (4-Ethylcatechol)	ethyl side-chain + hydroxyl instead of methoxy group
DMB	91-16-7	1,2-Dimethoxybenzene (Veratrol)	no side-chain + methoxy instead of hydroxyl group
DMT	494-99-5	1,2-Dimethoxy-4-methyl-benzene (3,4-Dimethoxytoluene; 4-Methylveratrol)	methyl side-chain + methoxy instead of hydroxyl group
DMS	6380-23-0	1,2-Dimethoxy-4-vinyl-benzene (3,4-Dimethoxystyrene; 4-Vinylveratrole)	vinyl side-chain + methoxy instead of hydroxyl group
ADB	93-15-2	4-Allyl-1,2-dimethoxybenzene (Methyleugenol)	methoxy instead of hydroxyl group
GUA	90-05-1	2-Methoxyphenol (2-Hydroxyanisole; Guaiacol)	no side-chain
MMP	93-51-6	2-Methoxy-4-methylphenol (Creosol)	methyl side-chain
EGU	2785-89-9	4-Ethyl-2-methoxy-phenol (4-Ethylguaiacol)	ethyl side-chain
MVP	7786-61-0	2-Methoxy-4-vinylphenol (4-Vinyl-guaiacol)	vinyl side-chain
MAN	100-84-5	1-Methoxy-3-methyl-benzene (3-Methylanisole)	methyl side-chain + no hydroxyl group
EAN	10568-38-4	1-Ethyl-3-methoxy-benzene (3-Ethylanisole)	ethyl side-chain + no hydroxyl group
VAN	626-20-0	1-Methoxy-3-vinyl-benzene (3-Vinylanisole)	vinyl side-chain + no hydroxyl group
EST	140-67-0	1-Allyl-4-methoxybenzene (4-Allylanisole; Estragole)	no methoxy group + methoxy instead of hydroxyl group

### Experimental protocol

Compounds listed in Table 1 were tested at different doses. The dose was calculated according to the substance purity as from the details provided by the supplier. For each dose, 10 bioassays against the stimulus to be tested and 10 bioassays against the negative control (in this case the arenas were treated with 100 µl of the solvent alone) were conducted. Each bioassay was run with a different tick in a different arena.

All the substances were tested at the doses of 100 and 1000 µg, corresponding to 6.37 and 63.65 µg/cm^2^ respectively. If a substance was found repellent at 100 µg, it was also tested at 10 µg and if repellent at 10 µg, it was tested at 1 µg.

Bioassays were all run at room temperature under daylight conditions. Temperature was monitored at the beginning and at the end of each experimental session; observed temperature variations were from 0.1 to 1.1°C. The minimum and maximum temperatures recorded in the whole period in which the bioassays were carried out were 21.1 and 28.7°C respectively; however, room temperature did not seem to affect the bioassay results.

### Statistical analysis of the results

Data from the bioassays were not normally distributed, therefore the median was used as a central tendency estimator and non parametric methods were used for hypothesis testing. In order to check for possible significant differences between treatments and negative controls, the time spent by ticks to move from line A over line B was compared using the nonparametric Mann-Whitney *U* test.

To represent the repellency of compounds, we used the ratio obtained dividing the median of time before crossing the finish line recorded in the bioassays against the stimulus by the median recorded in the bioassays against the negative control. Correlation between substances' repellency and vapor pressure was tested using Spearman's rank correlation coefficient, assigning a rank from 0 to 3 to the substances according to their repellency as follows: rank 3: compounds that are repellent at 10 µg (low dose); rank 2: repellent at 100 µg (medium dose); rank 1: repellent only at 1000 µg (high dose); rank 0: not repellent at any dose.

## Results

### Repellency of pure compounds

The bioactivity of the pure compounds tested in this study is reported in Table 2. Out of all the tested substances, 2 (MAN, EAN) were not repellent at all, 3 (GUA, DMB, EST) proved to be repellent only at the highest dose of 1000 µg, while 6 (MMP, EGU, SYR, DMT, DMS, VAN) were repellent also at the dose of 100 µg, this medium dose being the minimum at which eugenol was repellent in the previous study [23]. Five compounds (MVP, ADP, ADB, CAT, MCA) were repellent at the dose of 10 µg, that is the minimum dose at which DEET proved to be repellent with this bioassay [23]; remarkably, a substance (ECA) demonstrated a statistically significant difference between the negative control and the treatment even at the lowest dose of 1 µg.

**Table 2:** Bioactivity of pure compounds at different doses.

Stimulus	Dose (µg)	Treatment median (s)	Negative control median (s)	*P*	Treatment/negative control
EUG 1	1	17.5	24.0	n.s.	0.7
EUG 1	10	22.5	14.0	n.s.	1.6
EUG 1	100	128.0	9.5	<0.01	13.5
EUG 1	1000	192.5	12.0	<0.01	16.0
EUG 2	10	7.5	14.0	n.s.	0.5
EUG 2	100	182.0	9.5	<0.01	19.2
EUG 2	1000	193.5	12.0	<0.01	16.1
EUG 3	10	18.0	14.0	n.s.	1.3
EUG 3	100	80.0	9.5	<0.01	8.4
EUG 3	1000	187.0	12.0	<0.01	15.6
SYR	10	20.0	18.5	n.s.	1.1
SYR	100	184.0	18.5	<0.01	9.9
SYR	1000	195.5	18.0	<0.01	10.9
ADP	1	12.5	12.0	n.s.	1.0
ADP	10	166.0	16.5	<0.01	10.1
ADP	100	192.0	19.5	<0.01	9.8
ADP	1000	197.5	16.5	<0.01	12.0
CAT	1	12.0	12.0	n.s.	1.0
CAT	10	53.5	12.0	<0.01	4.5
CAT	100	193.0	12.0	<0.01	16.1
CAT	1000	193.5	9.5	<0.01	20.4
MCA	1	13.0	15.0	n.s.	0.9
MCA	10	195.5	15.0	<0.01	13.0
MCA	100	190.5	15.0	<0.01	12.7
MCA	1000	192.0	15.0	<0.01	12.8
ECA	1	16.5	9.0	<0.05	1.8
ECA	10	185.5	9.0	<0.01	20.6
ECA	100	192.5	9.0	<0.01	21.4
ECA	1000	196.5	9.0	<0.01	21.8
DMB	10	12.5	9.5	n.s.	1.3
DMB	100	12.5	9.5	n.s.	1.3
DMB	1000	196.0	9.5	<0.01	20.6
DMT	10	7.0	8.5	n.s.	0.8
DMT	100	106.0	8.5	<0.01	12.5
DMT	1000	199.5	8.5	<0.01	23.5
DMS	10	11.5	10.0	n.s.	1.2
DMS	100	188.5	12.5	<0.01	15.1
DMS	1000	200.0	10.0	<0.01	20.0
ADB	10	28.5	10.0	<0.05	2.9
ADB	100	184.5	16.5	<0.01	11.2
ADB	1000	194.0	10.0	<0.01	19.4
GUA	100	13.0	13.0	n.s.	1.0
GUA	1000	51.5	12.5	<0.01	4.1
MMP	10	10.5	12.0	n.s.	0.9
MMP	100	63.0	8.5	<0.01	7.4
MMP	1000	200.5	10.0	<0.01	20.1
EGU	10	11.5	10.0	n.s.	1.2
EGU	100	184.0	8.5	<0.01	21.6
EGU	1000	181.5	14.0	<0.01	13.0
MVP	10	22.0	10.0	<0.05	2.2
MVP	100	148.0	12.5	<0.01	11.8
MVP	1000	191.5	10.0	<0.01	19.2
MAN	100	17.0	24.5	n.s.	0.7
MAN	1000	8.5	14.0	n.s.	0.6
EAN	100	11.0	14.0	n.s.	0.8
EAN	1000	22.5	14.0	n.s.	1.6
VAN	100	16.5	8.5	<0.05	1.9
VAN	1000	182.5	10.0	<0.01	18.3
EST	100	14.0	9.0	n.s.	1.6
EST	1000	181.5	12.5	<0.05	14.5

“*P*” indicates the statistical significance of the difference between treatment and negative control (n.s.  =  no statistically significant difference). “Treatment/negative control” represents the ratio between the treatment and negative control medians. The results of three independent replicates of eugenol from [23] are reported as a reference (EUG 1, EUG 2, EUG 3).

To focus the attention only on the most active compounds and simplify further analysis, we considered as repellent, at a certain dose, only substances showing a ratio between the treatment and the negative control median of about 10 or higher (Table 2). According to this rule, 3 compounds were repellent at the dose of 10 µg (ADP, MCA, ECA), 7 at 100 µg (EGU, MVP, SYR, DMT, DMS, ADB, CAT; eugenol would fall into this class), 4 only at the dose of 1000 µg (MMP, DMB, VAN, EST) and 3 were not repellent at all (GUA, MAN, EAN). In the rest of the article, this classification will be used.

### Correlation between repellency and vapor pressure

**Figure 2:**
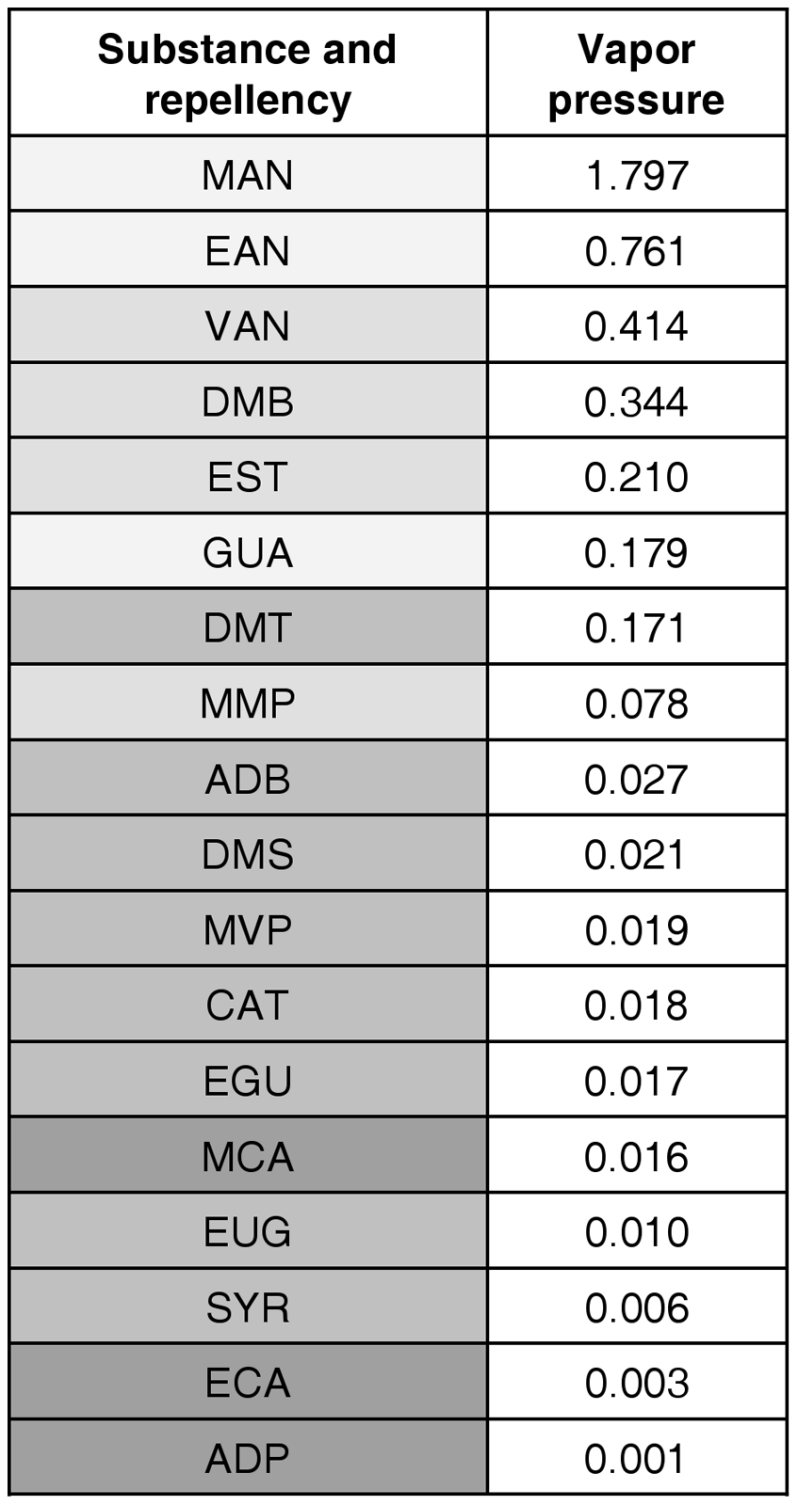
Repellency and vapor pressure (listed in descending order) of each tested compound. Grey tones indicate the repellency of each molecule: dark grey represents molecules that are repellent at the lowest dose (10 µg); medium grey, molecules repellent at the medium dose (100 µg); light grey, molecules repellent only at the highest dose (1000 µg); very light grey, molecules which are not repellent even at the highest dose. Predicted vapor pressure values (mmHg) at 25°C are from: ACD/Labs Predicted Properties (In: ChemSpider website. URL: <http://www.chemspider.com>, accessed 21Jan 2010).

Spearman's rank coefficient highlighted a statistically significant correlation between repellency and vapor pressure of the tested substances (*r_S_* = −0.8993; *P*<0.001); in particular, it appeared that the lower the vapor pressure, the higher the repellency (Figure 2).

### Correspondence between repellency and structure

**Figure 3:**
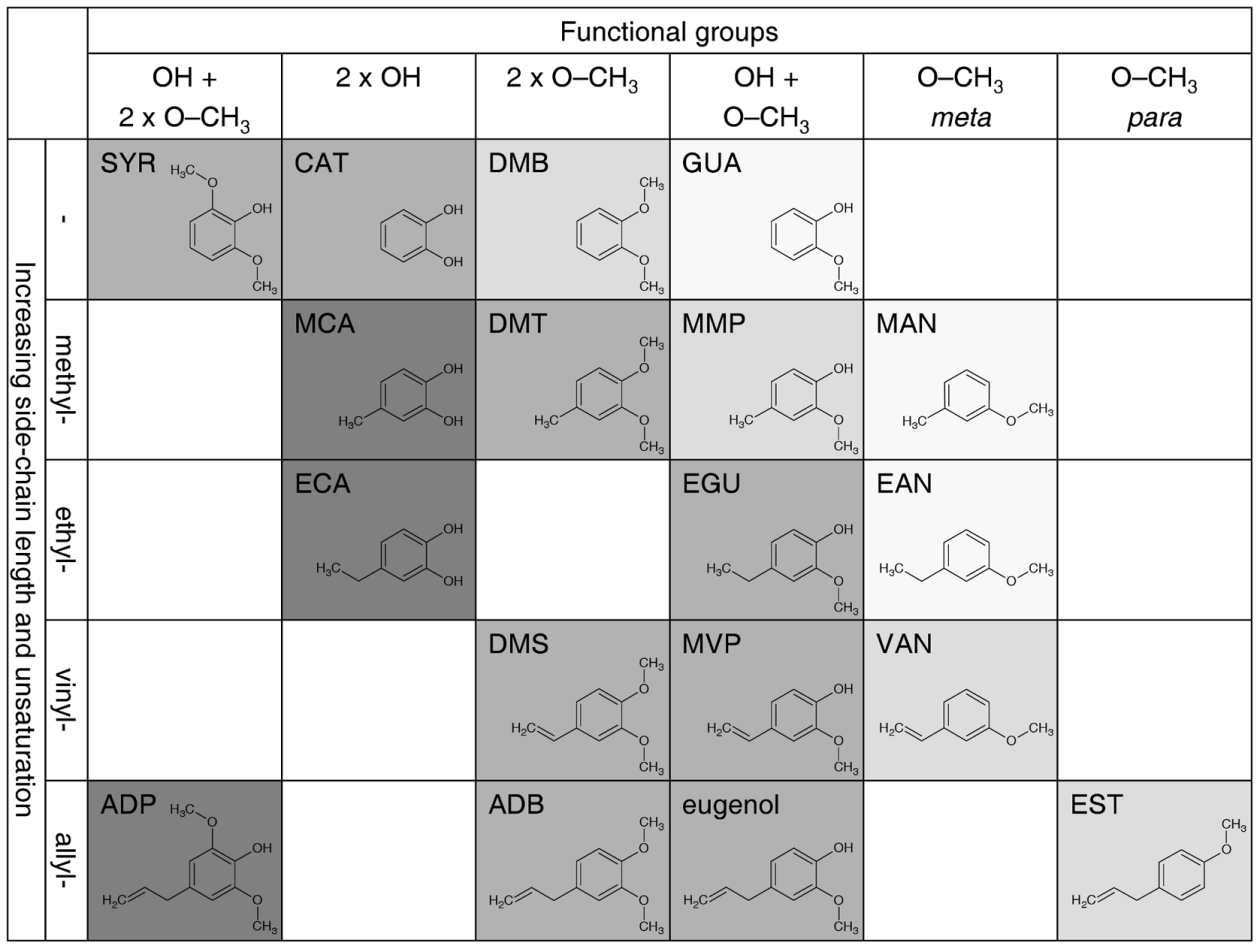
Graphical classification of the compounds tested in this study and repellency. Columns contain molecules with the same combination of functional groups (hydroxyl –OH and methoxy –O–CH_3_ groups), but with a different side-chain (no side-chain or methyl, ethyl, vinyl, allyl side-chains) on the benzene ring; rows contain molecules that have the same side-chain but different functional groups. Grey tones indicate the repellency of each molecule: dark grey represents molecules repellent at the lowest dose (10 µg); medium grey, molecules repellent at the medium dose (100 µg); light grey, molecules repellent only at the highest dose (1000 µg); very light grey, molecules which are not repellent even at the highest dose.

To highlight possible correspondences between repellency and molecular structure we used a 2-dimensional graph, where compounds are ordered according to the functional groups attached to the benzene ring and the length and saturation of the carbon side-chain; in the graph, repellency is rendered with increasing tones of a color so that darker areas mark combinations of chemical features that appear to have a stronger influence on repellency (Figure 3).

The compound classification in this artificial space pointed out the role of oxygen; in fact, it appeared that both the hydroxyl and methoxy groups were, at least partly, involved in repellency, since compounds having either 2 or 3 hydroxyl or methoxy groups (in various combinations) were more repellent than those carrying only 1 methoxy group. Moreover, compounds with 2 hydroxyl groups or with 1 hydroxyl and 2 methoxy groups showed the highest repellency.

The carbon side-chain also seemed to play a role: in fact, the longer the chain, the higher the repellency.

## Discussion

Many of the compounds tested in this study, which are all similar, to some extent, to the repellent eugenol, appeared to be active in the bioassay.

In principle, in the bioassay used here, contact between the tick's sensory apparatus and the treated area of the arena is possible. However, according to our observations, this happened only in a very few cases, strongly suggesting that olfaction rather than tasting is involved in the observed biological activity of tested compounds.

In general, the mode of action of repellents on blood feeding arthropods is still somehow controversial. Several different hypotheses have been postulated so far that could all be valid under different circumstances. For example, in the case of the widely used DEET, some Authors proposed a mechanism implying an interference with the recognition of attractive odors (host or food odors), rather than a real repellent activity of the substance itself [44], [45]. Similarly, Pellegrino et al. [12] considered DEET as a modulator of the general olfactory receptor activity, capable of disrupting the insect odor code. In contrast, other Authors suggested an effective avoidance-behavior induced by the recognition of the molecule by some specific arthropod olfactory receptors, irrespective of other stimuli (see [13], [14], [46] for examples regarding insects; [47] for ticks). Both hypotheses are probably valid under different conditions, as suggested by the results in [48], [49], where repellent molecules demonstrate, in different circumstances, either inhibitory or excitatory activities on olfactory receptors, by reducing agonist-evoked currents or eliciting currents in the absence of agonists, respectively. However, the avoidance behavior of the repellent treated area observed with this bioassay (see, for example, the track in Figure 1) is consistent with an effective repellency of the tested substances. Moreover, possible attractive olfactory stimuli coming from the observer should not play a major role in this case, since the observer always wore gloves and stayed at a distance during the bioassays; as a matter of fact, ticks moving in the arena did not show any preferential direction regardless of the observer's position.

Anyway, the relationship between the characteristics of a molecule and its biological activity against a certain arthropod species is not yet clearly understood, despite several attempts to correlate physical, structural and chemical properties of a molecule with its repellency have been made (see for example [40]–[42] and citations therein). Nevertheless, Paluch et al. [41] found that vapor pressure and electronic and electrotopological descriptors are important components in models describing the repellency of sesquiterpenes against mosquitoes; moreover, Davis ([50], cited in [51]) found that both oxygen functional groups and vapor pressure are important factors related to insect repellent activity.

As regards vapor pressure, we found that the lower the vapor pressure, the higher the repellency (Figure 2). Also the highly repellent substance DEET presents a vapor pressure of 0.001 mmHg at 25°C, that is comparable with that of the most repellent compounds tested here. As stated in a similar case study [43], the low or nil bioactivity of compounds with higher vapor pressure could not be a direct consequence of a lack of intrinsic repellency since it may result from a quick disappearance of the substances from the arena. However, this does not seem the case of our study, given the relatively low vapor pressure of all compounds tested here, so that the experimenter could perceive their odor coming from the arena throughout the assay and for a long time after the end of it, confirming the persistency of the substances. Like in [41], the observed negative correlation between vapor pressure and bioactivity could also be due to the bioassay setup, involving a static air design and a small space in relation to the size of the assayed animal. In fact, under these conditions, a lower vapor pressure allows the maintenance of a well-defined gradient between the treated and untreated areas in the bioassay environment. Conversely, large air flow-through systems could lead to a positive correlation between repellency and vapor pressure, since, in that case, the higher the volatility of the compound, the higher the amount that could possibly reach the arthropod's sensilla from the distance. In any case, the significant correlation between repellency and vapor pressure does not necessarily imply that the vapor pressure itself directly influences repellency, since vapor pressure is related to the molecular structure of the compound, which could be the major driver of repellency.

As for the chemical structure, the role of different moieties in the repellent properties of insect repellents has been investigated by several Authors (see for example [41], [42] and citations therein). In these studies, amides, imides, phenols, alcohols, hydroxy ethers, glycols and hydroxy esters were all shown to be involved in the repellent properties of the molecules. Moreover, within specific classes of molecules, repellency seemed to increase from acetate to the corresponding alcohol to the corresponding ester, and unsaturated alcohols were better repellents compared with saturated ones (effects of unsaturation are considered also in [43]). In general, several studies emphasized the importance of the oxygen (in particular of the hydroxyl –OH groups), that was confirmed here.

Some indication of a possible relationship between unsaturation and repellency was noted in the present study, by comparing, for example, the biological activity of EAN (saturated ethyl side-chain) and VAN (unsaturated vinyl side-chain). A similar relationship was reported also by Moore [52, cited in 42], who observed that unsaturated alcohols are more repellent than saturated ones. However, the case of EGU (saturated ethyl side-chain) and MVP (unsaturated vinyl side-chain), showing a similar repellency despite the presence of a double bond in the latter, suggests caution in drawing any definitive conclusions on this subject. In general, the importance of unsaturation can be confirmed, but the direction of this effect does not always seem clear, as also noted, for example, in [43], where an unsaturation in a certain position could increase or decrease the repellency depending on the molecules considered.

In any case, the data presented in this study confirm the strong influence of the molecular structure on the bioactivity of repellent compounds in ticks as already observed in other arthropods. In theory, this may be related to the variable volatility of different compounds, so that, for example, the presence of oxygen would not be important per se but for the effect that an oxygen atom can have on the vapor pressure of the compound. However, a vast body of evidence about the importance of steric and chemical interactions between signals and receptors in chemosensory systems points towards this direction.

In general, odorants are recognized by several olfactory receptors with distinct affinities and each receptor has a distinct odor-response profile [53]–[59]. Studies on the vertebrate olfactory system have demonstrated the existence of receptors activated by eugenol and structurally related substances. For example, the rat vanilloid receptor 1 is activated by eugenol and capsaicin (a molecule sharing the methoxyphenol moiety with eugenol) [60], and the so called “eugenol mouse olfactory receptor” (mOR-EG) recognizes eugenol and similar substances (EGU and MMP in the present work) [55]. It was also demonstrated that, despite mOR-EG has some tolerance for certain substitutions in the target molecule, it is however highly sensitive for other structural changes [55]. Noteworthy, in the same study, mOR-EG did not respond to guaiacol (GUA), a substance that did not show any repellency in the present study. Insects have also got olfactory receptors that respond to eugenol or structurally related compounds: for example, AgOr65 (in the mosquito *Anopheles gambiae* Giles) is responsive to eugenol and similar substances [54], [57], [61]; likewise, Or59a (in the *Drosophila* fly larvae) responds to methyleugenol (ADB in the present study) and to the similar anisole (methoxybenzene) and methylphenol [56]; methyleugenol (ADB) has also been shown to upregulate the odorant receptor co-receptor Orco in the *Bactrocera dorsalis* (Hendel) fly [62]. Unfortunately, only few studies have been carried out so far on tick olfactory receptors [32], [34]–[38]. For example, Leonovich [34] demonstrated the presence of phenol and lactone olfactory receptors in the distal sensilla of the *I. ricinus* Haller's organ. Altogether, the available evidences suggest that at least 1 receptor with high affinity for substances with molecular structure similar to that of eugenol could be present in ticks as well. In this case, such a receptor (or receptors) could well be involved in the recognition of the substances considered in this study.

In addition to olfactory receptors, odorant-binding proteins (OBPs) and chemosensory proteins (CSPs) are involved in the insect olfactory system activity. In general, each OBP can bind several ligands with different affinities, depending on the cavity volume and conformation and the residues of the binding site [63]–[66]; for example, eugenol proved to be the best ligand assayed for AmelOBP14, an *Apis mellifera* L. OBP [63], [65]. Unfortunately, the scarcity of data about the proteins involved in odorants recognition in ticks, strongly limits the potential of studies relating the chemical structure of ligands to the binding affinity of OBPs in these arthropods. In fact, in an exhaustive comparative genomic analysis of the odorant gene families in 20 arthropod species, only 1 CSP gene and no OBP and olfactory receptor genes (or even pseudogenes) were found in the whole *I. scapularis* genome (the only tick genome completely sequenced so far) [39].

Regardless the mechanisms accounting for the repellency of eugenol and related compounds against ticks, the question about the ultimate causes of such repellency remains open. In this respect, the acaricidal effects of some of these compounds (that could result, for example, from primordial arms races between herbivores and plants [67]) on other tick species may provide a possible clue [27].

In conclusion, despite the incomplete knowledge of tick's chemosensory system prevented a more detailed investigation, some molecular features influencing the bioactivity of tick repellents were identified in this study; this could allow, in the future, an easier comprehension of the underlying mechanisms of repellency. Under a practical point of view, the remarkable biological activity of some of the compounds tested here appears to be very promising even though further studies, investigating toxicology, persistence and efficacy under field conditions, will be necessary to confirm their potential as novel ingredients for tick repellents.
