# Correction: From Chemistry to Behavior. Molecular Structure and Bioactivity of Repellents against *Ixodes ricinus* Ticks

**DOI:** 10.1371/annotation/6636cea1-b3f2-4f93-acf7-b34c5aabce07

**Published:** 2013-07-08

**Authors:** Simone Del Fabbro, Francesco Nazzi

The name of the first author was given incorrectly. The correct name is: Simone Del Fabbro. 

The correct citation is: Del Fabbro S, Nazzi F (2013) From Chemistry to Behavior. Molecular Structure and Bioactivity of Repellents against Ixodes ricinus Ticks. PLoS ONE 8(6): e67832. doi:10.1371/journal.pone.0067832. 

